# Vertical preparation: a new technique for analogical and digital impressions

**DOI:** 10.4317/jced.60169

**Published:** 2023-07-01

**Authors:** Pablo Castelo-Baz, María Freire-Álvarez-Blázquez, Patricia Pereira-Lores, Pablo Álvarez-Nóvoa, Ana Dablanca-Blanco, Ramón Miguéns-Vila, Benjamín Martín-Biedma

**Affiliations:** 1 DDs, MsCa, PhD. Professor and Director, Master ENDORE, Department of Stomatology, School of Dentistry, University of Santiago de Compostela, Spain; 2 DDs, MsC. Clinical Professor, Master ENDORE, School of Dentistry, University of Santiago de Compostela, Spain; 3 DDs, MsC, PhD. Clinical Professor, Master ENDORE, School of Dentistry, University of Santiago de Compostela, Spain; 4 DDs, MsC, PhD. Professor and Director, Master ENDORE, Department of Stomatology, School of Dentistry, University of Santiago de Compostela, Spain

## Abstract

The BOPT technique (Biologically Oriented Preparation Technique) was proposed by Loi et al. (1) and has become a popular form of vertical preparation for complete crowns with promising results. In this procedure, the clinician can operate on the gingival tissue during preparation and modify its structure in thickness and height by adding modifications on the provisional restoration. However, one of the main challenges in this technique is the transference of information about the gingival tissue to the laboratory technician, who will place the margin of the restoration randomly in a space determined by two marks on the working cast. The technique proposed enables the accurate transmission of the exact point where the margin of the restoration needs to be placed. Furthermore, it facilitates the recording of conventional impression materials and intraoral scanners due to the compression of polytetrafluoroethylene (PTFE) tape into the sulcus, which allows to arrest bleeding or intracrevicular liquid and is easily registered irrespective of the method of impression used.

** Key words:**Vertical preparation, BOPT, PTFE, emergence profile, digital impression, conventional impression.

## Introduction

When preparing a tooth for a complete crown, two patterns have been described in literature regarding to the finish line: horizontal preparation (with a well-defined margin) and vertical preparation (without margin) (1). In vertical preparation there is no finish line. The laboratory technician positions the crown margin based on the information from the gingival tissue (1). Depending on the definitive position of the restoration regarding to the gingival margin, we distinguish between feather-edge preparations or BOPT preparations (1).

The BOPT technique (Biologically Oriented Preparation Technique) is one form of vertical preparation proposed by Loi in 2013 (1). In this technique, the clinician and the technician can work on the periodontium, modifying its shape, position and scallop regardless of the previous gingival situation or pre-existing margin, since the cemento-enamel line is erased (1,2). The advantages of this technique to a clinical and biological level are achieved through the restoration, both provisional and definitive, by adding modifications in the position of the margin, the emergence profile and on the shape of the tooth (1).

One of the challenges in BOPT technique is the location of the margin regarding to the position into the gingiva. The crown should never go beyond the apical limit of the sulcus. Therefore, the impression taken after the preparation (either analogue or digital) must give the laboratory technician the maximum information to properly locate the margin of the restoration. The most common method to reproduce the BOPT preparation and the shape of the gingival sulcus is the classic two-step analogic impression with double retraction cord (1,3), which presents a main drawback: the collapse of soft tissue during the impression, which can lead to errors in reproducing the position of the margin (3).

The objective of this article is to describe a simple, reproducible technique that can improve the transmission of the information on the exact position of the margin within the sulcus and can also simplify impression-taking for BOPT both with analogical and digital techniques.

## Case Report

-Patient 1: Analogical Impression

**Figure 1:**
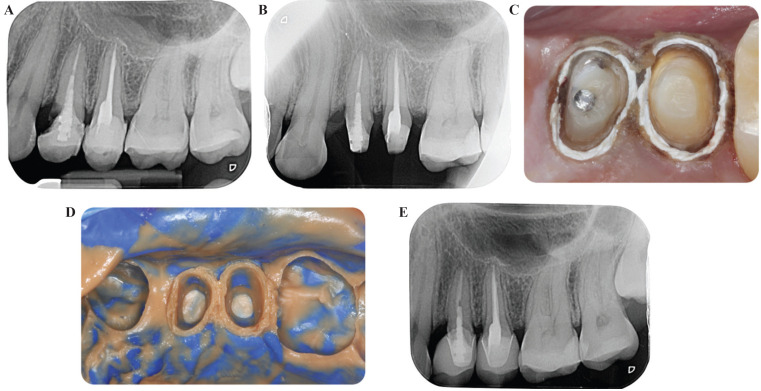
(A) Preoperative periapical radiograph. (B) Periapical radiograph of the reconstruction and preparations. (C) PTFE tape placed into the sulcus. (D) Impression of the preparations. (E) 1 year follow up of the definitive crowns.

A 42-year-old woman came to the clinic to improve the aesthetics of her upper left first and second premolars. During the clinical examination it was observed that both teeth showed large composite restorations entirely covering the clinical crowns. The upper left first premolar showed a carious lesion affecting the mesial aspect of the tooth right up to the bone crest. Radiographically, both teeth were endodontically treated teeth without signs of periapical pathology (Fig. 1A). A first periodontal probing was performed to determine the attachment level of the epithelium. Afterwards, the distance from the gingival margin to the bone crest was measured. The gingival tissue showed signs of inflammation but not signs of periodontal disease or bacterial plaque. There was no bone defect, and the cortical bone was intact.

Given the limited remaining structure, the mesial caries lesion and due to aesthetic requirements, the treatment plan consisted in a crown lengthening procedure followed by two BOPT complete crowns. The failing composite restorations were removed, the caries lesions were treated and both abutments were built-up.

Following the guidelines proposed by Loi (1) both teeth were prepared using the BOPT technique (Fig. 1B). Afterwards, two provisional restorations were made. The function of this provisional crown is to stabilize the blood clot caused after preparation, which will initiate the biologic response by stimulating cell differentiation and promoting the formation of new gingival tissue and a new periodontal structure that will adapt to the emergence of the provisional (1) To make the provisional restoration, Protemp 4 bisacrylic resin (3M ESPE, St Paul, MN, USA) and a silicone index (Virtual putty, Ivoclar Vivadent AG, Schaan, Liechtenstein) were used. The temporary restoration is then adapted and polished (1).

After 4 weeks, once the gum maturation time had taken place, the provisional crowns were removed and the health and position of the gingival tissue was checked. An impression was taken using the conventional two-step technique with additive silicone (Light Body and Virtual putty; Ivoclar Vivadent AG, Schaan, Liechtenstein). For impression-taking, a rolled piece of PTFE (polytetrafluoroethylene) tape was adapted, inserting it 1 mm subgingivally to imitate the position of the cementoenamel junction (CEJ) (Fig. 1C). This will prevent excess of impression material from flowing inside the sulcus and also allows total control of the depth of the margin throughout the 360º of the tooth. The insertion of the PTFE tape helps the clinician determine the exact depth at which the finish line should be located and the emergence profile of the restoration. Once the impression was taken, the presence of a well-defined and homogeneous margin was verified (Fig. 1D). Two monolithic lithium disilicate crowns (IPS E.max CAD, Ivoclar Vivadent AG) were fabricated and finally bonded with resin cement (Multilink, Ivoclar Vivadent AG, Schaan, Liechtenstein) (Fig. 1E).

-Patient 2: Digital Printing

**Figure 2:**
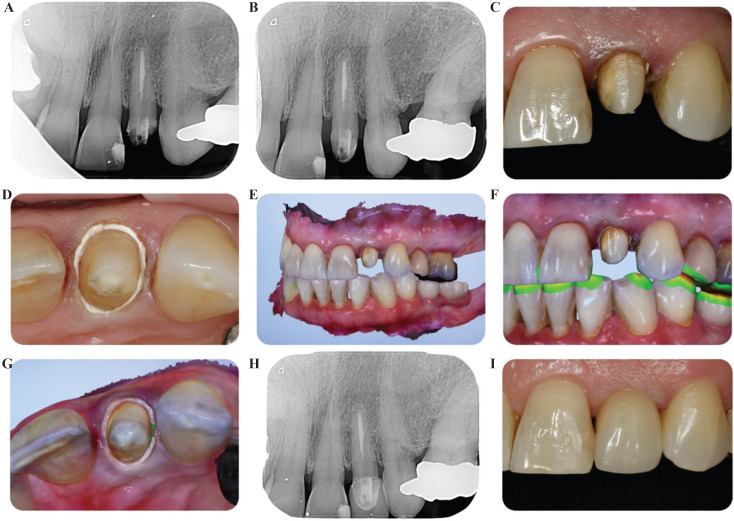
(A) Preoperative periapical radiograph. (B-C) Periapical radiograph and photograph of the preparation. (D) PTFE tape placed into the sulcus. (E-F) STL image of the scan. (G) STL image of the prepared tooth with the PTFE tape into the sulcus. (H-I) Periapical radiograph and final photograph with the bonded crown.

A 48-year-old woman came to the clinic with a coronal fracture of her upper left lateral incisor. Clinical and radiographical examination showed enough ferrule and no signs of periapical pathology (Fig. 2A). The patient had good gingival health, as well as an adequate level of epithelial attachment and bone crest level. The esthetic concerns of the patient, the remaining dental tissue and the good periodontal conditions leaded to the decision to prepare the remaining structure using the BOPT technique for a complete monolithic lithium disilicate ceramic crown (IPS E.max CAD, Ivoclar Vivadent AG). After preparation, (Fig. 2B,C) a provisional restoration was made with Protemp 4 bisacrylic resin (3M ESPE, St Paul, MN, USA).

4 weeks after preparation and placement of the provisional restoration, the maturation of the tissues was achieved. A digital impression was taken using the TRIOS Color Pod intraoral scanner (3Shape A / S, Copenhagen, Denmark). To overcome the limitations of intraoral scanners, when determining the finish line for subgingival preparations, a piece of PTFE tape was placed around the tooth. It was inserted 1 mm subgingivally to simulate the position of the CEJ and determine the depth of the margin of the future restoration (Fig. 2D). PTFE tape helps the scanner to register the finish line as it allows compaction into the sulcus to the exact level where the technician has to place the margin of the definitive restoration. It also allows to arrest bleeding, saliva or intracrevicular liquid, which can lead to errors in scanning. Once the STL files were obtained (Fig. 2E-F), a well-defined margin was observed around the preparation (Fig. 2G), which facilitated the CAM design of the future crown, eliminating the risk of invading the biological width and allowing greater accuracy when designing the margin. Finally, the crown was bonded with a resin cement (Multilink, Ivoclar Vivadent AG, Schaan, Liechtenstein) and occlusion was adjusted (Fig. 2H-I).

## Discussion

In vertical preparations, the prosthetic technician positions the margin according to the information provided by the gum tissue (1). Replicating the gingival tissue as faithfully as possible becomes crucial.

The BOPT technique allows the gingival tissue to adapt to the prosthetic form of the prepared tooth or implant abutment. The design of the emergency profile of the crown is crucial.

The classic impression method for BOPT preparations is the traditional technique with double retraction cord. However, the digital technique with intraoral scanner (IOS) has proved to be equally precise in capturing individual preparations, partial fixed dental prostheses with 3-4 elements or even full-arch restorations. Digital scanning is followed by CAD-CAM production of monolithic restorations or cad-restorations for veneering (3,4).

In both techniques, when the provisional prosthesis is removed, there is a collapse of the soft tissue. This causes a distortion between the real position of the gingival margin and the position captured by the impression material or the intraoral scanner (3).

The proposed technique is not the first attempt in improving control in positioning the margin of the BOPT restoration. Bauza *et al*. also propose a technique that requires two impressions for the fabrication of the definitive restoration. It is a laborious, time-consuming technique in which the whole process depends on the accuracy of the first impression. Any error in the first impression or in any step of the process will be transferred to the whole procedure (5).

The main advantage of PTFE tape ist that it is very moldable. It can be inserted deeper into the sulcus depending on where the clinician wants to place the margin of the restoration. PTFE is highly compressible and can also be modified in its horizontal thickness if needed. It can also be used to mark very clearly the scallop of the gingiva at the interproximal aspect, which is very difficult to reproduce by the impression materials. When using a conventional impression technique, silicones reproduce its position with great clarity. Also digitally, the scanner can identify the position marked by the PTFE tape with great accuracy.

The technique described above avoids the imprecision of placing randomly the termination line between the gingival margin and the depth of the sulcus. The possibility of compacting the PTFE tape to the exact desired depth provides the technician the advantages of horizontal preparations and is simple and feasible. The clinical and biological advantages of BOPT preparations are also obtained.

## Conclusions

In BOPT technique, when the provisional prosthesis is removed, the gingiva collapses towards the tooth, making it difficult to obtain a digital impression of the preparation, the sulcus or its emergence. The BOPT technique is characterized by placing a termination line on the prosthesis itself, at a depth of 0.5-1 mm within the sulcus. There is some degree of inaccuracy in this space for the technician to position the margin of the crown. This technique allows to position the margin of the restoration in the exact desired position in a predicTable way both with analogical or digital impression.
